# *De novo* circular genome assembly of *Elizabethkingia anophelis* found in the mosquito *Aedes aegypti* from an Australian colony

**DOI:** 10.1128/MRA.00310-23

**Published:** 2023-08-25

**Authors:** Igor Filipović, Gordana Rašić

**Affiliations:** 1 The University of Queensland, School of Biological Sciences, St. Lucia, Australia; 2 Population Health, QIMR Berghofer Medical Research Institute, Mosquito Genomics, Herston, Australia; Indiana University, Bloomington, Bloomington, Indiana, USA

**Keywords:** *Elizabethkingia*, *Aedes aegypti*, long-read genome assembly, diversity

## Abstract

We report the complete circular genome assembly of *Elizabethkingia anophelis* (Flavobacteriales) generated with the ONT and Illumina sequences from a laboratory-reared *Aedes aegypti* mosquito. This genome sequence does not belong to the lineage of known isolates from *Anopheles* mosquitoes, indicating that *E. anophelis* is genomically diverse across mosquito disease vectors.

## ANNOUNCEMENT

*Elizabethkingia anophelis* (Flavobacteriales) is a Gram-negative bacillus (1) prevalent in the gut microbiomes of mosquitoes with a putative role in the mosquito-pathogen interactions (2) and is also considered as an emerging human pathogen (3). The type strain, R26^T^, was isolated from the midgut of the malaria mosquito *Anopheles gambiae* (4, 5), and the genome sequence of this and other *Anopheles* isolates forms a single lineage distinct from the human-derived isolates (3). Here, we report the complete genome sequence of *E. anophelis* from a major arboviral vector, mosquito *Aedes aegypti,* and show it is divergent from the isolates found in malaria vectors.

Total DNA was extracted from one *Aedes aegypti* male (NCBI SAMN33833734) from an Australian colony at QIMR Berghofer MRI, using a SPRI-bead protocol with DNA elution in 50  µL of TE buffer, which was used to generate both ONT and Illumina data. Four ONT libraries were prepared (each with 250 ng of DNA) using the Ligation Sequencing Kit SQK-LSK109 and sequenced on four R9.4.1 flow cells using the MinION device and the MinKNOW Software (ONT), yielding a total of 20.1 Gb with the read N50 of 16.1 kb. The base-calling was computed with Guppy v6.0.1 (ONT) using the super-accuracy configuration and long reads with ≥Q7; the long-read-based assembly was done with Flye v2.9 (6), using the minimum overlap between two reads of 10,000 bp (--min-overlap 10,000) and a metagenome mode (--meta). The draft assembly was polished with Pilon v1.23 (7) using the high-quality Illumina reads; a PCR-free Tagmentation library (Illumina 20041794) was prepared with ~20 ng of DNA and sequenced on one NextSeq 550 lane, producing 86.3 Gb of 300 bp paired-end data. Trimmomatic v0.39 (8) was used to remove adapters and retain ≥Q20 reads that were mapped to the draft assembly with BWA-mem (9) in default mode.

**Fig 1:**
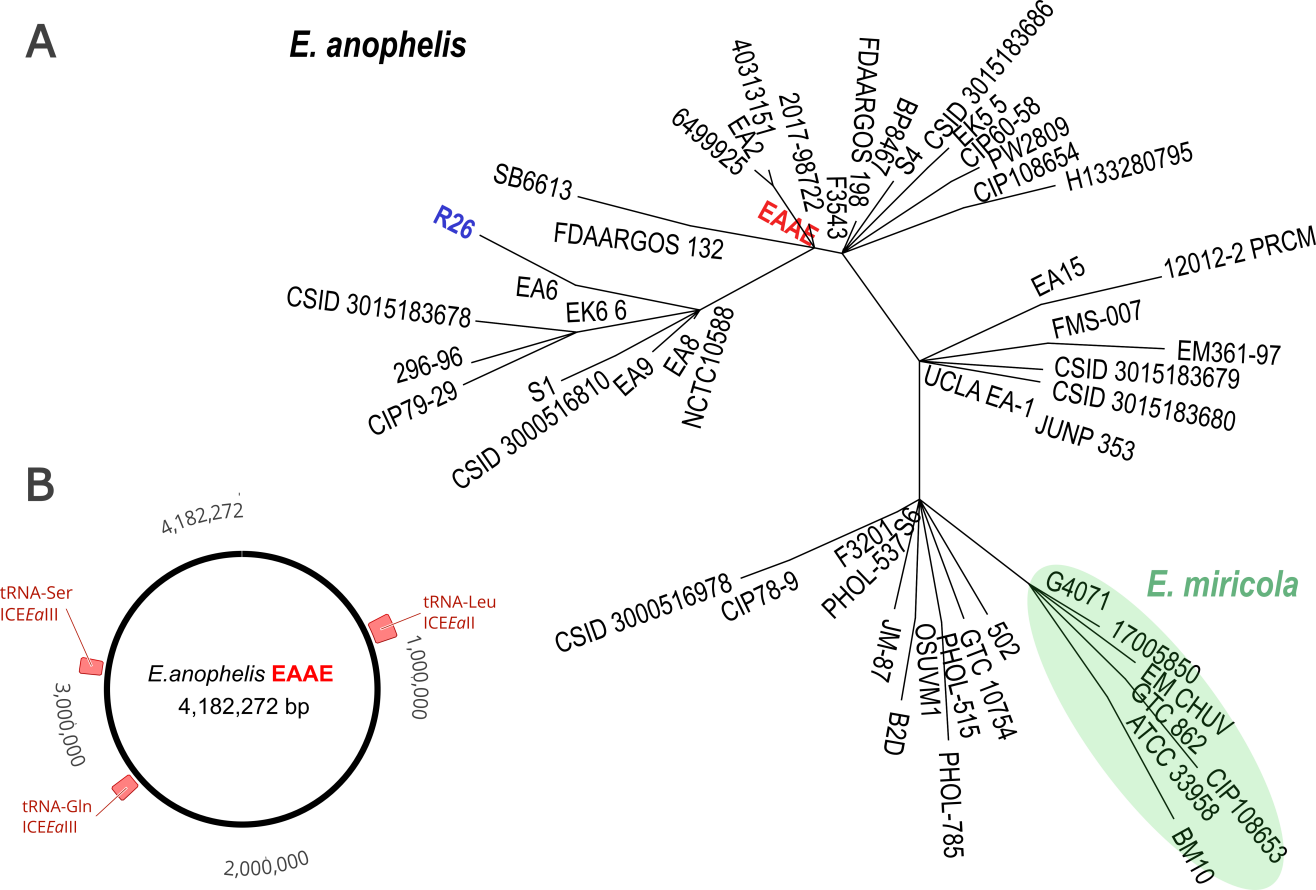
(A) Minimum spanning tree based on the unique cgMLST profiles at 1,546 panel loci in *Elizabethkingia anophelis* and *E. miricola* sequences from the Elizabethkingia PasteurMLST database (https://bigsdb.pasteur.fr/elizabethkingia/). The profile of the sequence from *Aedes aegypti* (EAAE in red) is distinct from the lineage that contains the type strain (R26 in blue) and other isolates from *Anopheles* mosquitoes. (B) The EAAE genome contains type II and type III integrative and conjugative elements that integrate next to a specific tRNA gene: tRNA-Leu ICE*Ea*II has 99.97% nucleotide identity (coverage 51%) with BK010601 (13), tRNA-Gln ICE*Ea*III has 91.46% identity (coverage 97%) with BK010612 (13), and tRNA-Ser ICE*Ea*III has 99.45% identity (coverage 71%) with BK010606 (13).

*E. anophelis* genome sequence was identified as a full circular contig 4,182,272 bp in length with 98.25% average nucleotide identity [OrthoANIu (10)] to the reference R26 strain (NCBI GCA_002023665.2) and a depth of coverage of 16× from the ONT reads and 88× from the Illumina reads. *De novo* annotation in DFAST with default parameters (11) rotated the circular sequence to fix the origin at the *dnaA* gene and revealed 3,789 CDSs, 15 rRNAs, 52 tRNAs, and no CRISPR loci. The core genome multilocus sequence typing (cgMLST) at 1,546 panel loci (3) revealed 116 new alleles compared to 254 sequences in the Elizabethkingia PasteurMLST database (https://bigsdb.pasteur.fr/elizabethkingia/). The minimum spanning tree [MSTreeV2 in GrapeTree (12)] with unique cgMLST profiles revealed that the *Ae. aegypti* isolate does not belong to the lineage of *Anopheles* isolates (Fig. 1A). BLASTn search against the annotated sequences of integrative and conjugative elements (ICEs) in *E. anophelis* (13) showed that the sequence from *Ae. aegypti* contains type II and type III ICEs (Fig. 1B), unlike the sequences from *Anopheles* mosquitoes that have only type III ICEs (13). Overall, the uncovered genomic distinctiveness of *E. anophelis* from *Ae. aegypti* warrants broader sequence characterization of this bacterium across mosquito vectors.

## Data Availability

The annotated genome sequence of the *A. aegypti-*derived *Elizabethkingia anophelis* is deposited under the NCBI GenBank accession no. CP121098.1 and BioProject PRJNA930912. The ONT sequences (SRR24965245-SRR24965248) and Illumina sequences (SRR24965242) are available within the BioProject PRJNA946909.
